# WTAP regulates Mitochondrial damage and Lipid oxidation in HCC by NOA1 mediated m6A modification

**DOI:** 10.7150/jca.102618

**Published:** 2025-01-01

**Authors:** Sheng Liu, Mei Shang, Jiao Gong, Hengchang Sun, Bo Hu

**Affiliations:** 1 Department of Laboratory Medicine, Third Affiliated Hospital of Sun Yat-sen University, Guangzhou 510630, People's Republic of China.; 2 Guangdong Key Laboratory of Liver Disease Research, Third Affiliated Hospital of Sun Yat-sen University, Guangzhou 510630, People's Republic of China.

**Keywords:** WTAP, NOA1, Mitochondrial damage, Lipid oxidation, HCC

## Abstract

**Background:** Hepatocellular carcinoma (HCC) is one of the leading causes of cancer-related death worldwide. However, the molecular mechanism underlying the occurrence and development of HCC remains unclear. We are interested in the function of m6A methylation enzyme WTAP in the occurrence and development of HCC.

**Methods:** Expression of the m6A methylation-associated enzymes in paired carcinoma and adjacent tissues (N=17) were detected by RT-PCR. Electron microscopy was adopted to observe the subcellular organelle. GPX4 levels in hepatoma cells were analyzed by Western blot and RT-PCR. The Fe^2+^ and GSH/GSSG levels were detected using the corresponding kits. Mass spectrometry (MS) was conducted to determine the altered protein types in hepatoma cells. Finally, methylated RNA immunoprecipitation (MeRIP) was used to analyze the m6A methylation of Nitric oxide-associated protein 1 (NOA1).

**Results:** RT-PCR showed that there were no significant differences among tumor tissues and normal tissues in METTL3 (*p*=0.6485), FTO (*p*=0.1158), ALKBH (*p*=0.6148), YTH N6-Methyladenosine RNA binding protein F1 (YTHDF1) (*p*=0.3171), and YTH N6-Methyladenosine RNA binding protein F2 (YTHDF2) (*p*=0.1116). However, compared to normal tissue, WTAP (*p*=0.0011), METLL14 (*p*=0.0044) and YTH N6-Methyladenosine RNA binding protein F3 (YTHDF3) (*p*=0.0472) were obviously decreased in tumor tissues. The decrease of WTAP was most apparent. Conditional knockout of WTAP in Huh-7 and SNU-449 cells could induce mitochondria damage, which was manifested in smaller mitochondria and a compressed intermembrane space of mitochondria. The result was also confirmed by electron microscopy. Additionally, Huh-7 and SNU-449 cells with WTAP knockdown presented low mitochondrial membrane potential, while WTAP overexpression could reverse this effect. Interestingly, data from flow cytometry by Annexin V-FITC/PI and detection of pyroptosis-related marker Gasdermin D (GSDMD) by Western blot demonstrated that, overexpressing or knocking down WTAP will not affect cell apoptosis and pyroptosis in hepatoma cells. Furthermore, mRNA and protein levels of the key indicator GPX4 of ferroptosis in Huh-7 and SNU-449 cells with WTAP knockdown or overexpression were analyzed by RT-PCR and Western blot. It was shown that knockdown of WTAP promoted expressions of GPX4 in these cells (p<0.0001), but a distinct downregulation of GPX4 levels occurred in the WTAP overexpressing cells. Further study indicated that a significantly increase of GSH/GSSG levels and clearly decrease of Fe^2+^ concentrations appeared in Huh-7 and SNU-449 cells with WTAP knockdown (p<0.05). Opposite results were observed in the cells with WTAP overexpression (p<0.05). Moreover, we also clarified the effect of WTAP on modulating GSH synthesis might be independent of SLC7A11, not SLC3A2 or the Xc-system. Finally, mass spectrometry results showed that NOA1 might be related to WTAP. qPCR, WB and MeRIP-qPCR also confirmed WTAP regulated the m6A methylation of NOA1. It is supposed that NOA1 might be the molecule at the heart of the regulation mechanism by WTAP.

**Conclusion:** WTAP may affect the m6A methylation of NOA1 to induce mitochondrial damage, meanwhile activate the GPX4-axis to inhibit the lipid oxidation, resulting in the development of HCC.

## Introduction

Hepatocellular carcinoma (HCC), as the malignant tumor, is a major cause of cancer-related death. Owing to the high reported mortality and seriously inferior 5-year overall survival rate, HCC has become a major public health concern 1. Early symptoms of HCC are not obvious and difficult to diagnose. Most patients have advanced liver cancer at initial diagnosis, and treatment at this stage is limited and not curative. The only effective treatment is surgical resection or liver transplantation 2, 3. In clinical practice, Sorafenib is the first-line drug for advanced liver cancer but is prone to drug resistance and treatment failure 4. The pathogenesis of HCC remains largely unclear. Understanding the molecular mechanism of HCC and finding new therapeutic targets are important for clinical diagnosis and treatment.

Ferroptosis is a recently discovered non-apoptotic form of regulated cell death, mainly caused by the large production of iron-dependent lipid peroxides 5. Overwhelming evidence substantiates that it is involved in neurodegenerative diseases, tissue ischemia-reperfusion injury and tumors 5-7. Ferroptosis is also reportedly activated by reactive oxygen species (ROS) through the accumulation of lipid peroxide (PL-PUFA-OOH) 8. Glutathione peroxidase 4 (GPX4)-dependent glutathione (GSH) can reduce PL-PUFA-OOH, thereby inhibiting ROS-mediated ferroptosis 9. In addition, metabolic stress caused by cystine deficiency can lead to ferroptosis 10. Adenosine 5'-monophosphate (AMP)-activated protein kinase (AMPK) inhibits ferroptosis by restraining acetyl-CoA carboxylase (ACC) and reducing the synthesis of polyunsaturated fatty acids 11. Ferroptosis suppressor protein-1 (FSP1) is similar to GPX4; the inhibitory effect of FSP1 promotes cancer cell sensitivity to chemotherapy mediated by ferroptosis 12. The expression of prominin2 in response to ferroptosis stimulation can protect cells from ferroptosis 13. Activation of GPX4-induced ferroptosis may be a therapeutic strategy for tumors. However, the sensitivity of different tumor cells to GPX4 inhibitors varies greatly, indicating that other factors determine the resistance of tumor cells to ferroptosis 14.

There are many modifications of RNA in eukaryotes, such as the common 5 ' Cap structure and the 3 ' ploy A tail modifications are very important in transcriptional regulation 15. Common modifications of mRNA include N6-adenylate methylation (m6A), N1-adenylate methylation (m1A), and cytosine hydroxylation (m5C). Importantly, m6A methylation modification accounts for 80% 16. m6A methylation is involved in many physiological processes, such as affecting the splicing of mRNA precursors and the stability of mRNA, regulating the nuclear output of RNA and mRNA translation, and promoting the translation of circRNA by modification 17. What's more, researchers found that m6A methylation could change the development of tumors, regulate the directional differentiation of hematopoietic stem cells, and be involved in spermatogenesis 18, 19.

m6A methylation modification is reversible and is composed of methyltransferase writers, demethylase erasers and methylated reading proteins. Among these, the main components of methyltransferase complexes include wilms tumor 1 associated protein (WTAP), methyltransferase 3 (METTL3), methyltransferase 14 (METTL14), KIAA1492, etc. Their main roles are to catalyze the m6A modification of adenylate on mRNA. Demethylases include fat mass and obesity-associated protein (FTO) and AlkB homolog 5 (ALKHB5), which function to remove base methylation. It has been reported that demethylases FTO and ALKBH5 can demethylate m6A via an α-ketoglutarate and Fe^2+^-dependent form. Moreover, the YTH protein family has an m6A binding pocket domain in its three-dimensional structure that allows direct recognition 20, 21.

Nitric oxide-associated protein 1 (NOA1) is a nuclear encoded guanosine triphosphate (GTP) binding protein that predominantly localizes to mitochondria which stimulates oxidative phosphorylation (OXPHOS) activity 22-24. NOA1 regulates mitochondrial protein biosynthesis by influencing mitochondrial ribosome biogenesis 25, which is also confirmed by studies on the bacterial NOA1 homolog YqeH 26, 27. NOA1 could bind and potentially transport G quadruplex RNA to protect cells from staurosporine-mediated apoptosis 28.

This study is to clarify the role and potential mechanism of WTAP involved in the occurrence and development of HCC. Firstly, it was found that the expression of WTAP, as one of m6A methyltransferase, dramatically decreased in tumor tissues compared to normal tissue. Further experiments confirmed that WTAP could induce mitochondrial damage, inhibit GPX4 expression and Fe^2+^ concentration, improve the GSH/GSSG level, and induce lipid oxidation. MS results showed that NOA1 might be a potential target of WTAP in hepatoma cells. Consistently, RT-PCR displayed mRNA level of NOA1 changed with WTAP. In a nutshell, WTAP affected NOA1 to activate the GPX4 expression and reduce the Fe^2+^, improving GSH/GSSH levels to suppress ferroptosis of hepatoma cells, which may promote the occurrence and development of HCC. WTAP can be potentially novel target for the development of an alternative oncotherapy approach.

## Methods and Materials

### Patients

All the patients who were pathologically diagnosed with hepatocellular carcinoma (HCC) were enrolled in this study. None of these patients had received percutaneous ablation or chemo-embolization or radiotherapy before surgery. The inclusion criteria were as follows: (1) participants with HCC diagnosed by clinical or histological diagnosis; (2) HCC with a tumor diameter ≤5cm; (3) Non-lymph node spreading and no metastasis. The main exclusion criteria were as follows: (1) complicated with other tumors; (2) history of cancer resection surgery; (3) incomplete clinical data. The adjacent normal tissues were at least 2 cm from the matched tumor tissue. For all cases, paired tumor and adjacent normal tissue samples were obtained immediately after resection and were snap frozen in liquid nitrogen. All tissues were stored at -80°C until use. Paired non-tumor and tumor samples were collected from 17 HCC patients from the Third Affiliated Hospital of Sun Yat-sen University. Demographics and clinical characteristics of HCC specimens were listed in Table 1.

### Cell culture

The hepatoma cell lines Huh-7 (CCTCC SCSP-526) and SNU-449 (ATCC CRL-2234™) were cultured in DMEM supplemented with 10% FBS (PAN, Argentina) at 37°C in a 5% CO_2_ incubator (Thermo, Germany). siRNA-WTAP was purchased from Suzhou Jima gene Co., Ltd. The plasmid pcDNA3.1-WTAP was constructed, and siRNA and pcDNA3.1-WTAP were transfected into hepatoma cells with Lipofectamine™ 3000 Transfection Reagent (Invitrogen, German). The sequence of siRNA control, SiWTAP-1 and SiWTAP-2 are listed in the Table S1. In the 12 wells plate, 3μL siRNA (20μM) was mixed with 2μL Lipofectamine™ 3000 in 200μL Opti-MEM in one well, or 1μg plasmid mix with P3000^TM^ in 100μL Opti-MEM, 2μL Lipofectamine™ 3000 in 100μL Opti-MEM, 5 mins later, mixed the plasmid and Lipofectamine™ 3000 fluid gently, added the complex into one well in the 12 wells plate. The sequences of primers used in this study were listed in Table S2.

### RT-PCR

The tissues were ground by a tissue homogenizer (Servicebio, Wuhan, China) with a sterile steel ball (60Hz, 30 sec on, 30 sec off, 30 sec, on, 3 times) in 1 mL TRIzol (Invitrogen, Carlsbad, CA, USA). Cells were washed with PBS 2 or 3 times and dissolved in 1 mL Trizol reagent. Total RNAs were extracted using TRIzol reagent (Invitrogen, Carlsbad, CA, USA). First-strand cDNA was synthesized from the total RNA using the ReverTra Ace-First strand cDNA Synthesis Kit (Toyobo, Osaka, Japan) according to the manufacturer's instructions. RT-PCR was conducted in a 96-well microtiter plate (GeneStar, USA) with an LightCycler480 Real-Time PCR system (Roche, USA) using the following conditions: 95°C for 5 min, 35 cycles of 95°C 10 s, 55°C 20 s and 72°C 20 secs, the fluorescence signals generated were recorded during each PCR cycle. The mRNA samples were quantified using RT-PCR with the SYBR Green real-time PCR Master Mix kit (Roche, USA). β-actin mRNA was used as a reference housekeeping gene for normalization. The fold change was calculated as 2-ΔCt, where ΔCt = Ct (experimental group) - Ct (control group). All experiments were performed at least three separate times. The sequences of primers used in this study were listed in Table S2.

### Plasmids, antibodies, and reagents

WTAP gene was amplified from the cDNA of SNU-449 or Huh-7 and cloned into pcDNA3.1-Myc-His. The siRNA was synthesized by JiKai biotechnology Inc (Jiangsu, China). The following antibodies (Abs) were used in this study: anti-human GPX4 Rabbit mAb (ab125066, Abcam, UK), anti-human SLC7A11 Rabbit mAb (#12691, Cell Signal Technology, USA), anti-human SLC3A2 Rabbit pAb (abs100601, Absin, China), anti-human NOA1 Rabbit pAb (103652-T32, Sino Biological, China), anti-human GSDMD Rabbit pAb (abs143289, Absin, China), Anti-human m6A antibody (No.202003, Synaptic Systems, Germany), anti-human β-actin Recombinant antibody (81115-1-RR, Proteintech, USA), anti-Rabbit IgG HRP-linked Antibody (#7074, Cell Signal Technology, USA), Peroxidase AffiniPure Goat Anti-Mouse IgG (H+L) (SA00001-1, Proteintech, USA).

### Western blot

Huh7 or SNU-449 cells were washed with PBS three times and incubated with RIPA buffer on ice for 30min, cells were treated three times with an ultrasonic cell crusher, the concentration of total protein was determined by BCA kit. Cell lysates were mixed with 5×SDS loading buffer and boiled at 100°C for 8 min. The homogenized proteins were separated SDS-PAGE and transferred to PVDF membranes. The PVDF membrane was blocked by 5% milk in TBST at 37°C for 1 hour, then incubated with the primary antibody diluted with TBST at a 1:1000 overnight at 4°C. After washing with TBST four times, they were incubated with horseradish peroxidase-linked secondary antibodies (1:5000 diluted with TBST) at 37°C for 1 hour. After washing four times, the bands were detected using an Electrochemiluminescence (ECL) Imaging System. Image J was used for semi-quantitative analysis.

### Electron microscopy

Electron Microscopy was used as previously described 29. Briefly, SNU-449 cells in control groups and WTAP knocking-down or overexpression groups were washed with PBS three times, scraped immediately, then centrifuged at 1500rpm for 5min, fixed in 3% phosphate-glutaraldehyde. They were post-fixed, embedded, cut, and mounted at the Electron Microscopy Core Facility (Department of Anatomy, University of Louisville, KY, USA). Ultrathin sections were analyzed using a Hitachi H-7500 transmission electron microscope (Hitachi, Tokyo, Japan).

### Mitochondrial membrane potential detection

Hepatoma cells (SNU-449 and Huh7 cells) were seeded in 6 well plate and treated with siRNA or overexpressed plasmid, 48 hours later, cells were washed with PBS for three times, then added 0.5ml JC-1 dyeing working solution (Beyotime Biotechnology, China), incubated in the incubator at 37°C for 20 min. The cells were washed once with 1 to 3 ml of iced JC-1 staining buffer for twice, covered with an appropriate amount of JC-1 staining buffer, observed with fluorescence microscope (Nikon, Japan). CCCP (10 mm) is added to the cell culture medium in the ratio of 1:1000 as positive control.

### Flow cytometry

For assessing the apoptosis level, SNU-449 cells were treated with siRNA or overexpressed plasmid, 48 hours later, cells were washed with PBS for three times, the apoptosis rate was detected by the Annexin V FITC / PI apoptosis detection kit (Keygen Biotech, China) and analyzed by flow cytometer Beckman CytoFLEX FCM (Beckman, USA).

### Fe^2+^ concentration detection

Huh-7 and SNU-449 cells were transfected with siRNA or pcDNA3.1-WTAP. 48 hours later, the cells were washed three times and resuspended in 150 μL PBS. all cells were rapid-frozen and thawed 5 times. After centrifugating at 1500rpm for 10min, supernatants were detected using the Iron assay Kit (DiaSys Diagnostic Systems, Germany) in an automatic biochemical analyzer (Hitachi, Tokyo, Japan).

### GSH/GSSG and LPO assay

GSH/GSSD and lipid hydroperoxide (LPO) concentrations in Huh-7 and SNU-449 cells were measured according to the commercial kit instructions (Jiancheng Bioengineering Institute, Nanjing, China).

### Liquid Chromatography/Mass Spectrometry (LC/MS)

LC-MS/MS was conducted as previously described 30. A total of 100 mg homogenized sample mixed with 1 mL cold methanol/acetonitrile/H_2_O (2:2:1, v/v/v), then sonicated at a low temperature (30 min/once, twice), centrifuged for 20 min (14,000 g, 4°C). The supernatant was dried in a vacuum centrifuge. Dried sample was dissolved in 100 µL acetonitrile/water (1:1, v/v), adequately vortexed, and then centrifuged (14,000 rpm, 4°C, 15 min). The supernatants were collected for LC-MS analysis.

### Methylated RNA immunoprecipitation-PCR (MeRIP-qPCR) analysis

MeRIP-qPCR analysis was conducted as previously described 31. Briefly, after SNU-449 cells were treated with siRNA or overexpress plasmid, RNAs of experimental groups were acquired. mRNA was fragmented using RNA Fragmentation reagent (Invitrogen, AM8740) at 70°C for 15 min. A small amount of fragmented RNA was used as input RNA. Fragmented RNA was immunoprecipitated with anti-m6A antibody coupled to Dynabeads (Invitrogen, 10002D) in immunoprecipitation buffer (10 mM Tris-HCl, 150 mM NaCl, 0.1% Igepal CA-630 [Sigma-Aldrich, I8896] and 400 U RNasin Plus RNase inhibitor [Promega, N2611]) at 4°C for 2 h. m6A containing mRNAs were eluted twice with 6.7 mM N6-methyladenosine 5ʹ-monophosphate sodium salt (Sigma-Aldrich, M2780) at 4°C for 1 h and precipitated with 5 mg glycogen (Life Technologies, AM9510), one-tenth volumes of 3 M sodium acetate (Sigma-Aldrich, S7899) in 2.5 volumes of 100% ethanol at -80°C overnight. m6A enrichment was determined by RT-PCR analysis. The sequences of primers used are presented in Table S2.

### Statistical analysis

Data were presented as the mean ± SD and analyzed using GraphPad Prism V.5.00 software (GraphPad Software, San Diego, CA, USA). Differences between groups were assessed using an unpaired Student's t-test (two-tailed). One- or two-way analysis of variance (ANOVA) with Tukey's post hoc multiple comparison test was used to compare the means across multiple groups. All data met the assumptions of the statistical tests. Two-sided *p*-values<0.05 were statistically significant (*p < 0.05, **p < 0.01, ***p < 0.001, ****p < 0.0001).

## Results

### mRNA level of WTAP decreased in tumor tissues compared to normal tissue

**Figure 1:**
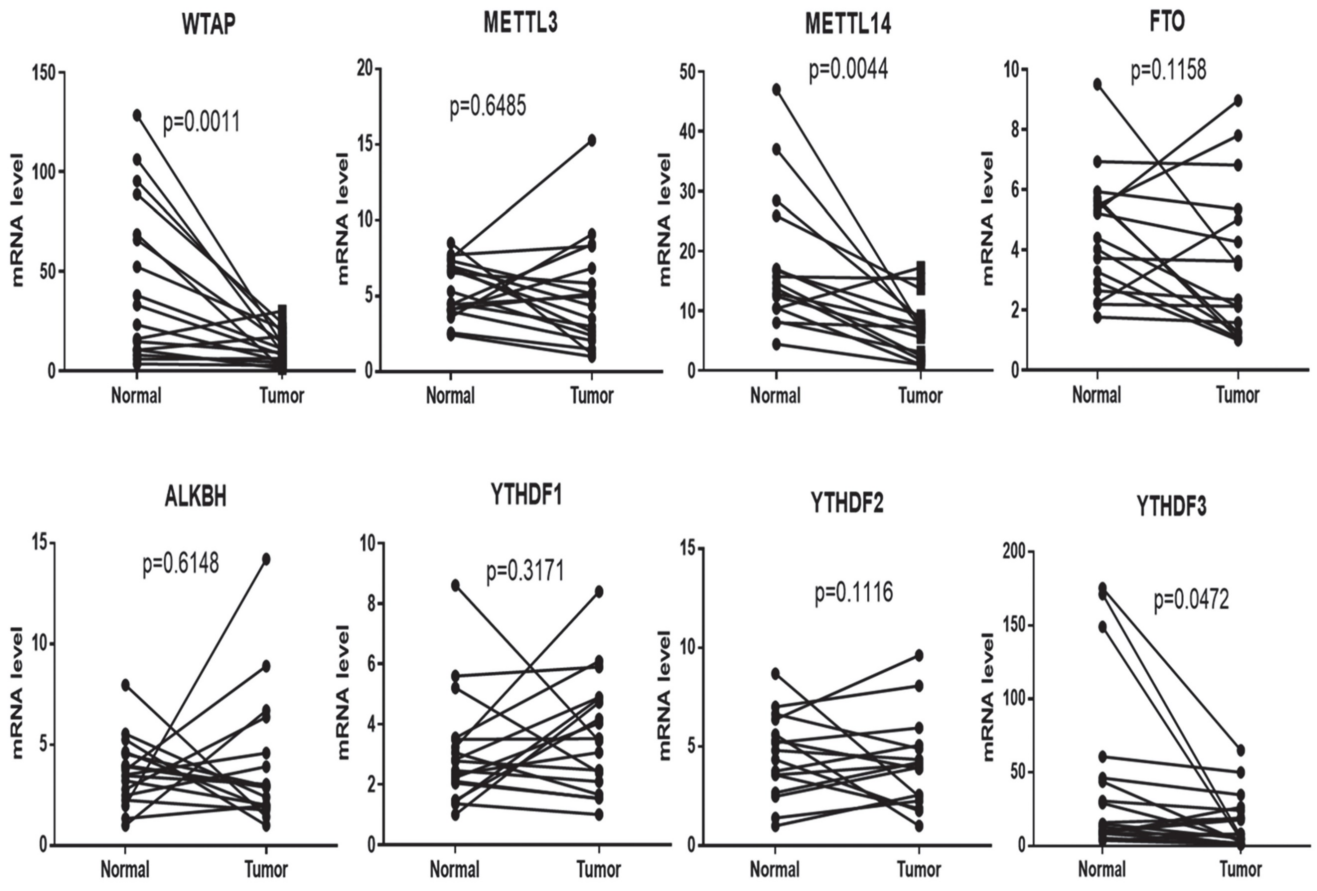
** mRNA expression of WTAP is decreased in tumors compared to normal tissues.** 17 pairs of tissues (tumor and normal tissues) were collected. The total RNA was extracted by TRIzol. RT-PCR was used to detect the mRNA levels of genes related to the m6A methylation complex. Paired t-test was used to analyze the results. Compared with normal group.

To explore m6A methylation change in the livers of HCC patients, 17 samples of tissues (Tumor and normal tissues) were collected; the total RNA was extracted by TRIzol, and qPCR was used to detect mRNA levels of the m6A methylation complex, including WTAP, METTL3, METTL14, FTO, ALKBH, YTHDF1, YTHDF2 and YTHDF3. All results showed that WTAP (*p*=0.0011), METLL14 (*p*=0.0044) and YTHDF3 (*p*=0.0472) were decreased in tumor tissues compared to normal tissue, meanwhile, there are no significant difference among the two groups in METTL3 (*p*=0.6485), FTO (*p*=0.1158), ALKBH (*p*=0.6148), YTHDF1 (*p*=0.3171), and YTHDF2 (*p*=0.1116) (Figure 1).

### Mitochondrial damage was observed in hepatoma cells with lower expression of WTAP

**Figure 2:**
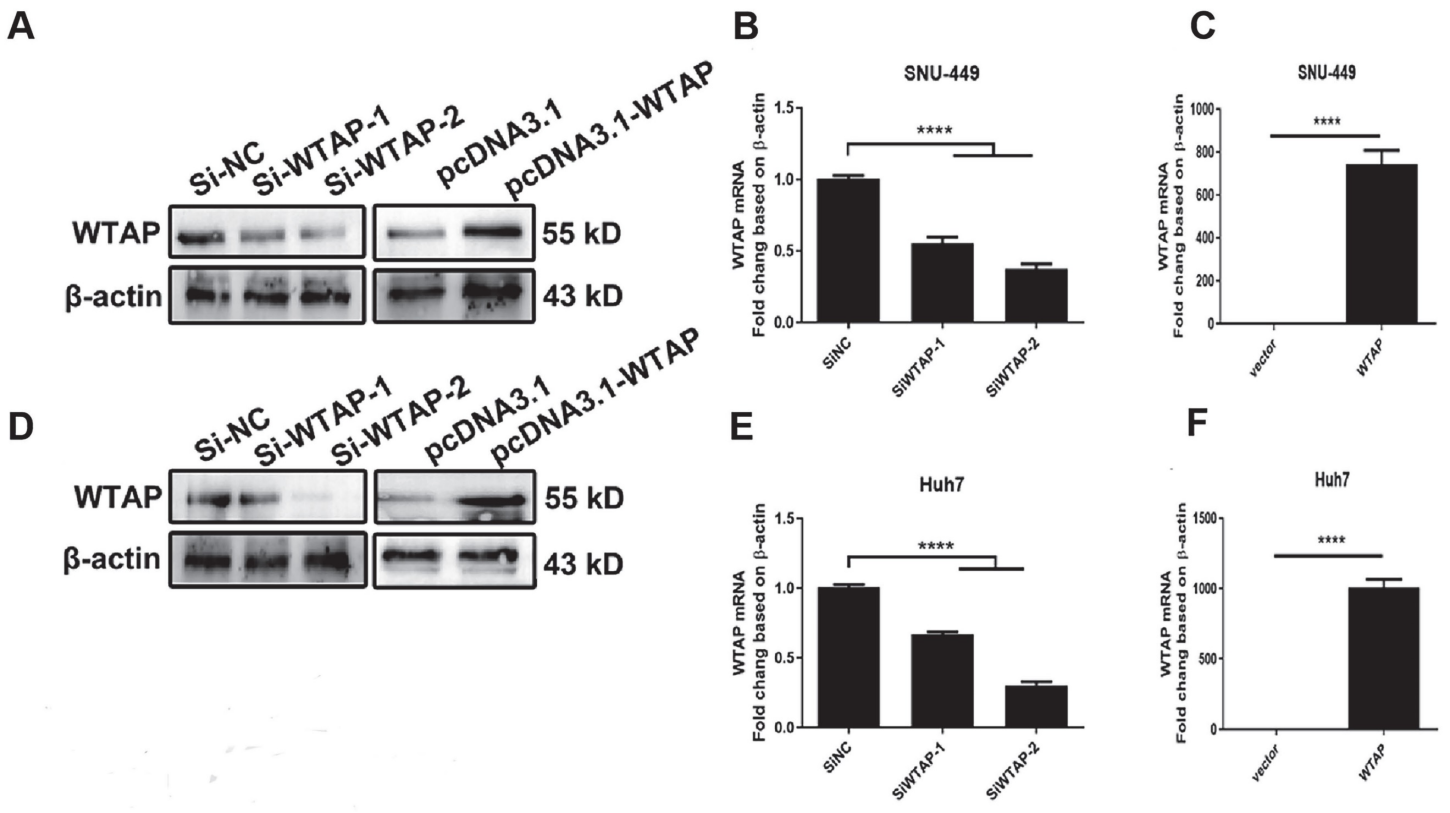
** Overexpressed plasmid and siRNA were used to regulate WTAP expression.** Two siRNAs of WTAP were designed and synthesized. The overexpressed plasmid pcDNA3.1-WTAP was constructed. Huh-7 and SNU-449 cells were transfected with the siRNAs or plasmid by Lipofectamine™ 3000. 48h later, the total RNA or cell lysates were collected. Western blot (**A, D**) and RT-PCR (**B-C, E-F**) were used to detect the mRNA and protein levels of WTAP. *p < 0.05, **p < 0.01, *** *p* < 0.001, ****p < 0.001 compared with control groups.

**Figure 3:**
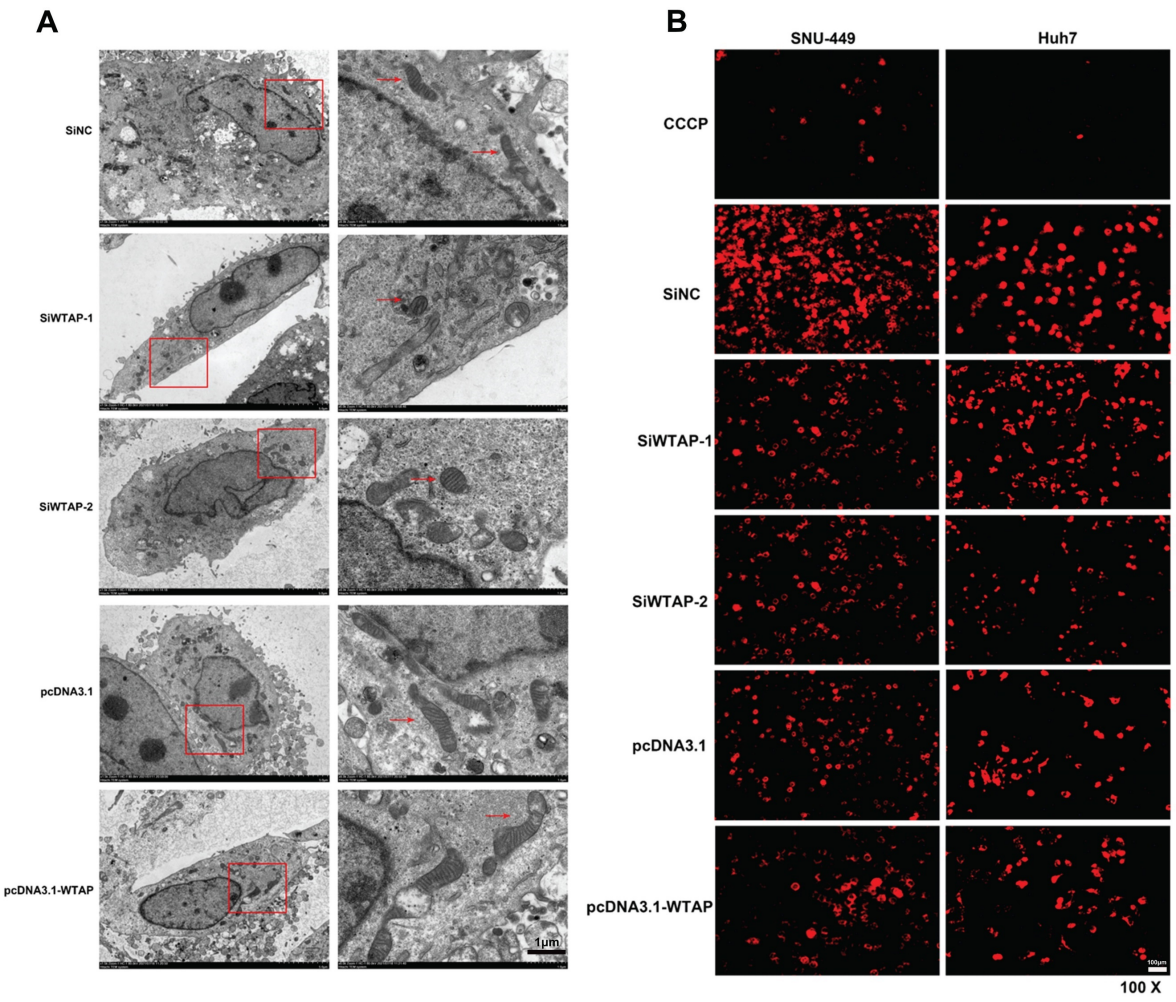
** Mitochondrial damage occurred in SNU-449 treated with siRNA of WTAP.** SNU-449 cells were transfected with the siRNAs or plasmid with Lipofectamine™ 3000. (A) 48h later cells were collected and fixed, then electron microscopy was used to view the mitochondrial morphology, scale bar=5μm (left), scale bare=1μm (right). (B) JC-1 was used to detect the mitochondrial membrane potential, scale bar=100μm, CCCP (10 mM) is added to the cell culture medium in the ratio of 1:1000 as positive control. CCCP, Carbonyl cyanide 3-chlorophenylhydrazone, a proton carrier (H+ ionophore), is a potent uncoupler of mitochondrial oxidative phosphorylation that induces permeability of the inner mitochondrial membrane to H+, leading to a loss of membrane potential on both sides of the inner mitochondrial membrane.

Studies show that m6A RNA methylation regulates mitochondrial function. To further confirm the effect of the RNA methyltransferase WTAP on mitochondrial homeostasis, we used siRNA to knockdown WTAP and pcDNA3.1-WTAP to overexpress WTAP in SNU-449 (Figure 2A-C) and Huh-7 cells (Figure 2D-F). Electron microscopy was used to observe the morphological change of organelles in SNU-449 cells by knockdown or overexpression of WTAP. It was shown that in the knockdown group, the mitochondria became smaller, while the intermembrane space of mitochondria was compressed (Figure 3A). It was suggested that lower WTAP damaged the mitochondria. Electron microscopy showed that mitochondrial function was impaired during WTAP knockdown. In addition, WTAP could affect mitochondrial membrane potential. Lower WTAP was associated with lower mitochondrial membrane potential, while higher WTAP could increase the mitochondrial membrane potential (Figure 3B).

### WTAP downregulated the GPX4 expression to regulate the lipid oxidation in hepatoma cells

**Figure 4:**
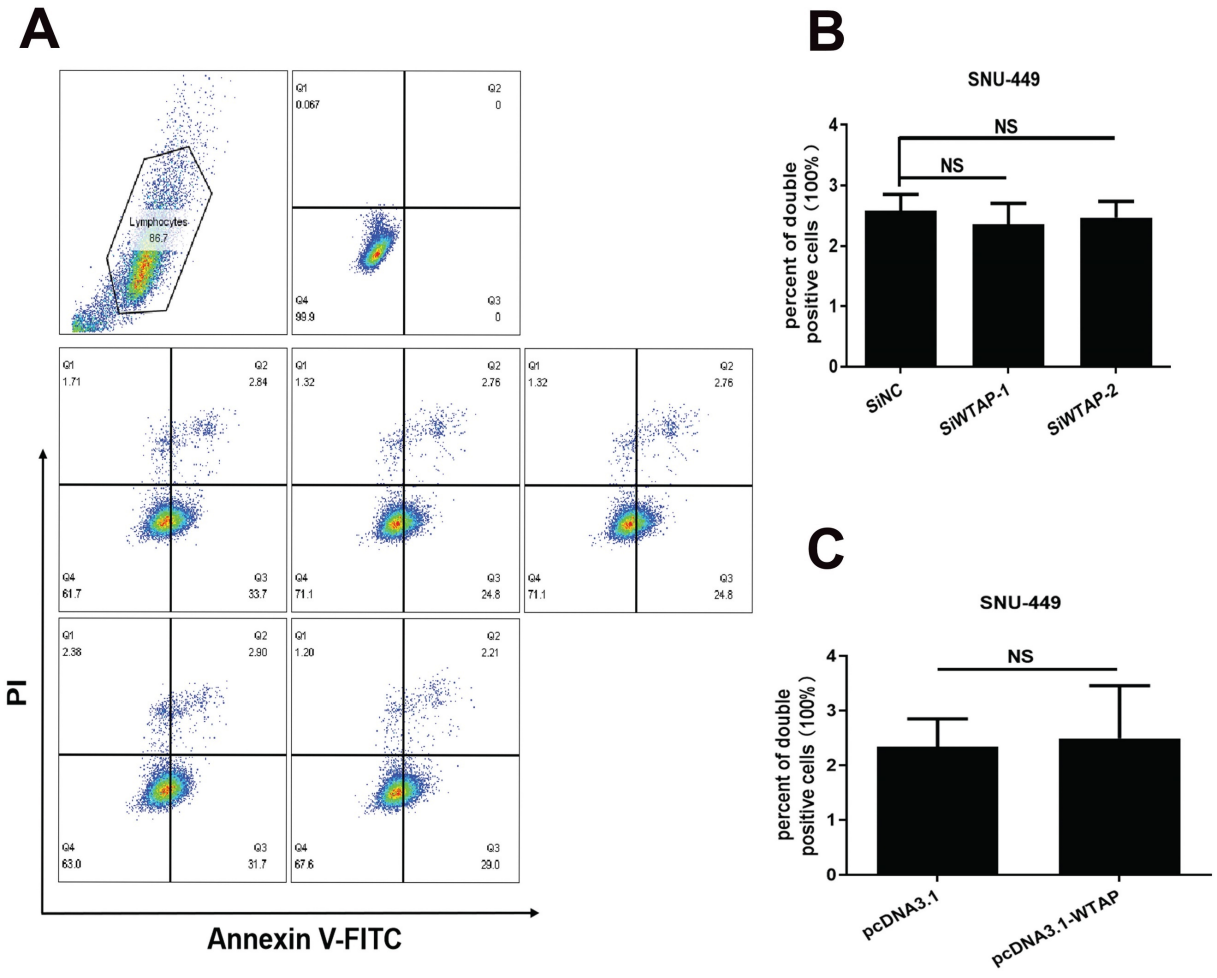
WTAP is independent of the apoptosis of SNU-449 cells. The siRNAs and plasmid were co-transfected into SNU-449 cells by lipo3000. 48h later, cells were digested with trypsin and stained with Annexin V-FITC and PI using the apoptosis detection kit, then cells were analyzed by flow cytometry. (A) Representative flow cytometry images. (B) The apoptosis level of SNU-449 cells transfected with siRNA. (C) The apoptosis level of SNU-449 cells transfected with pcDNA3.1 and pcDNA3.1-WTAP. *p < 0.05, **p < 0.01, *** *p* < 0.001, ****p < 0.001 compared with control groups; ns, no significance.

**Figure 5:**
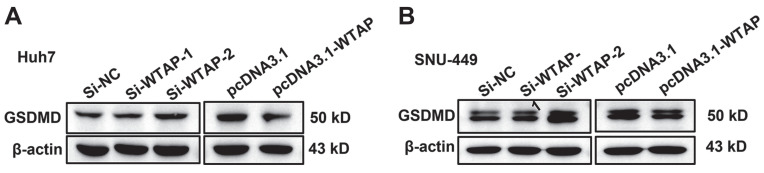
** WTAP is independent of the pyroptosis of Huh-7 and SNU-449 cells.** The siRNAs and plasmid were co-transfected to Huh-7 (A) and SNU-449 (B) cells by Lipofectamine™ 3000. 48h later, cell lysates were collected and WB was used to detect the expression of GSDMD, which was activated in pyroptosis.

**Figure 6:**
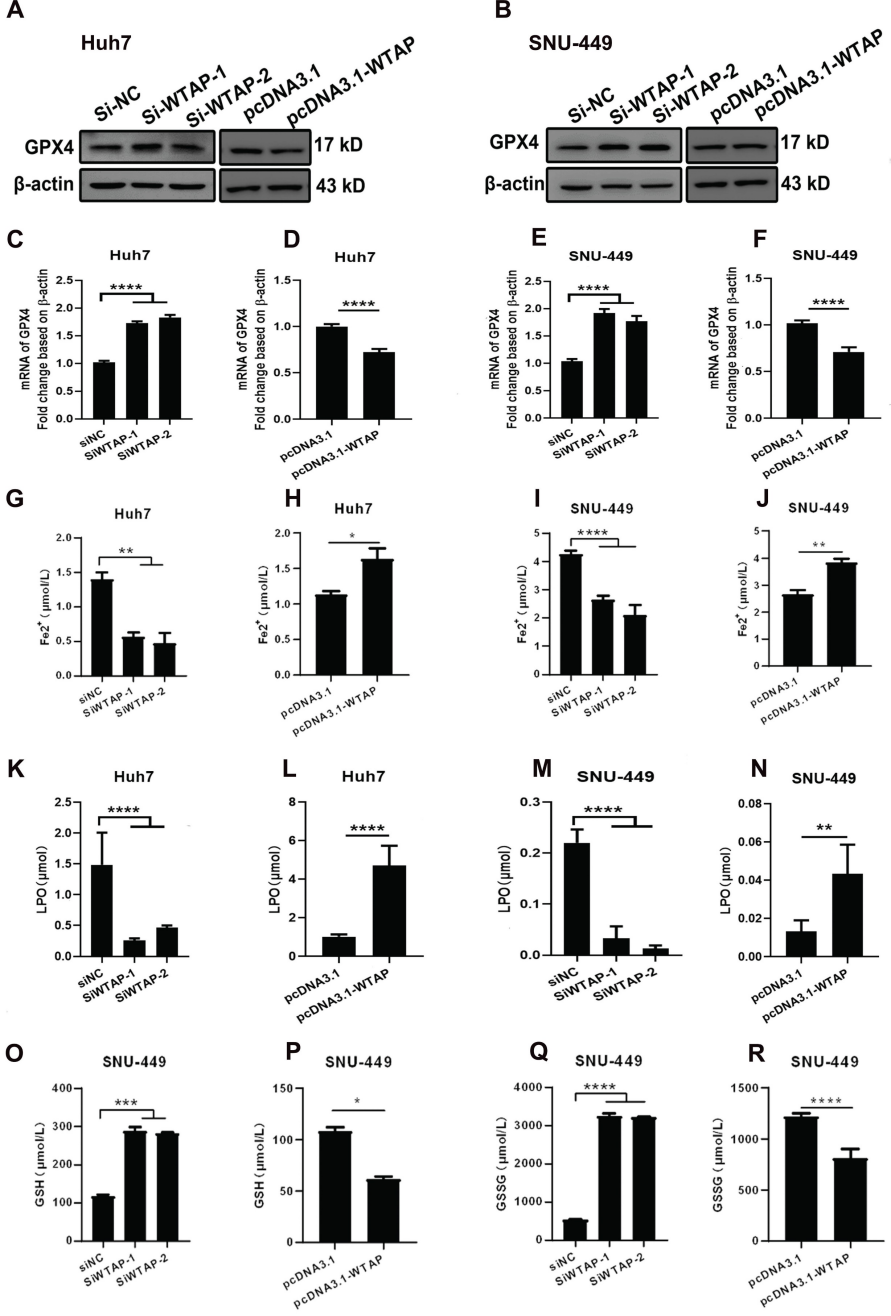
** WTAP is related to GPX4 expression to regulate the lipid oxidation in hepatoma cells.** Huh-7 and SNU-449 cells were transfected with the siRNAs or plasmid by Lipofectamine™ 3000. 48h later, total RNA or cell lysates were collected, RT-PCR and Western blot were used to detect the mRNA and protein levels of GPX4 (A-F). Fe2+ (G-J), LPO (K-N), GSH (O-P), and GSSG (Q-R) levels were detected. *p < 0.05, **p < 0.01, *** *p* < 0.001, ****p < 0.001 compared with control groups.

As we know, mitochondrial damage probably results in the occurrence of lipid oxidation, which has a close connection with ferroptosis. To verify this, the morphological change of hepatoma cells after treatment with siRNA of WTAP and analyzed cell death were observed. Compared with SiNC group, overexpressing or knocking-down WTAP in SNU-449 would not affect cell apoptosis, which reflected that there was no significant difference in percentage of Annexin V-PI double positive cells between control groups and WTAP overexpression or knockdown groups (*p*>0.05, Figure 4). Interestingly, further experiments presented that over-expression or knock-down of WTAP in Huh7 or SNU-449 cells would not change GSDMD levels (Figure 5). It is estimated that WTAP expressions in Huh7 or SNU-449 cells have no direct connection with cell apoptosis and pyroptosis. Recent studies have found that ferroptosis is involved in the development of many diseases, including hepatocellular carcinoma 32. To uncover the effect of WTAP on ferroptosis, we detected the levels of ferroptosis-related marker GPX4. Western blot and RT-PCR analysis revealed that as cells were transfected with siRNA to knockdown WTAP, an increase of GPX4 expression occurred in Huh-7 and SNU-449 cells. In contrast, overexpression of WTAP decreased GPX4 expression in SNU-449 and Huh-7 cells (Figure 6A-F). GPX4 is a well-recognized selenoprotein that can specifically catalyze glutathione to convert lipid peroxides into lipoids. It plays an important role in regulating ferroptosis. Ferroptosis is a unique form of cell death driven by iron-dependent phospholipid peroxidation, which is regulated by various cellular metabolic pathways, including redox homeostasis, iron metabolism, mitochondrial activity and metabolism of amino acids, lipids, and sugars, as well as various disease-related signaling pathways. It was shown that Fe^2+^ decreased in Huh-7 and SNU-449 cells (Figure 6G-J). Ferroptosis also could be activated by ROS through the accumulation of lipid peroxide (PL-PUFA-OOH). The LPO and GSH/GSSG levels were detected in Huh-7 and SNU-449 cells. Miraculously, LPO expression changed in the same direction with overexpression or knock-down of WTAP accompanied by the same direction, but changes in the opposite direction took place in the expressions of GSH and GSSG (Figure 6K-R). Studies demonstrated that the Cys-glutamic acid reverse transporter is an important link in ferroptosis, composed of SLC7A11 and SLC3A2. Glutamate can be transferred extracellularly through the cystine-glutamate reverse transporter system.

**Figure 7:**
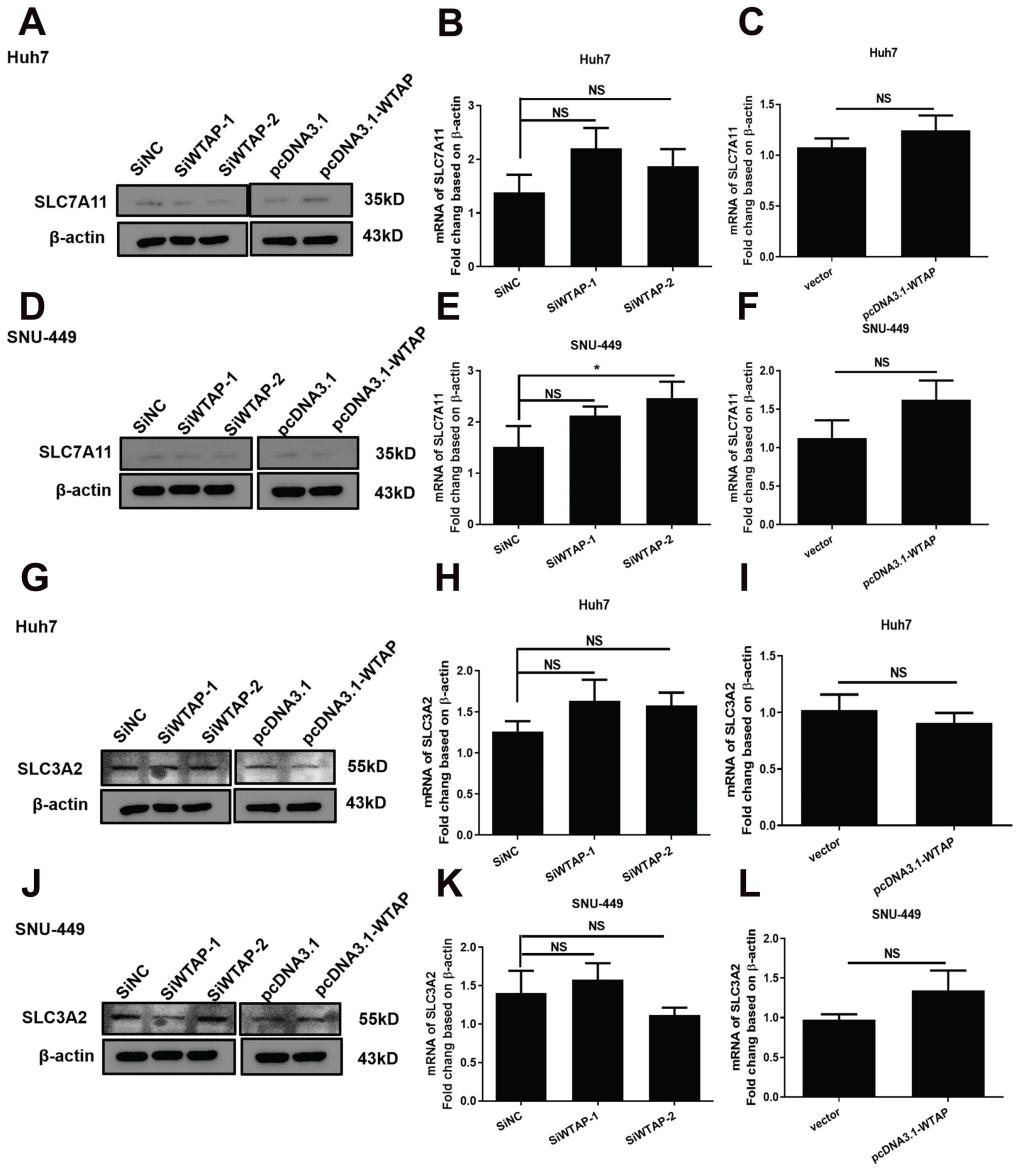
** WTAP is independent of SLC7A11, not SLC3A2 or Xc^-^ system in Huh-7 and SNU-449 cells**. The siRNAs or plasmid were co-transfected to Huh-7 and SNU-449 cells by Lipofectamine™ 3000. 48h later, the total RNA or cell lysates were collected. RT-PCR and Western blot were used to detect the mRNA and protein levels of SLC7A11 (A-F) and SLC3A2 (G-L). *p < 0.05 compared with control groups.

During extracellular cystine transfer into cells, cystine is reduced to cysteine, which is involved in the synthesis of GSH. As the synthesis of GSH increases, toxic lipid ROS levels decrease, exerting a protective effect against ferroptosis. The experiments showed that WTAP knockdown downregulated protein levels of SLC7A11 in Huh7 cells, but increased mRNA levels of SLC7A11 in SNU-449 cells. Overexpressing WTAP increased the protein levels of SLC7A11 in Huh7 cells, although there were no obvious differences in SNU-449 cells. For SLC3A2, there were no apparent changes among control groups, knocked-down groups, or overexpressed groups (Figure 7). All data suggested that WTAP does not affect the Xc^_^ system to affect GSH synthesis.

### WTAP regulated expression of NOA1 by m6A methylation

**Figure 8:**
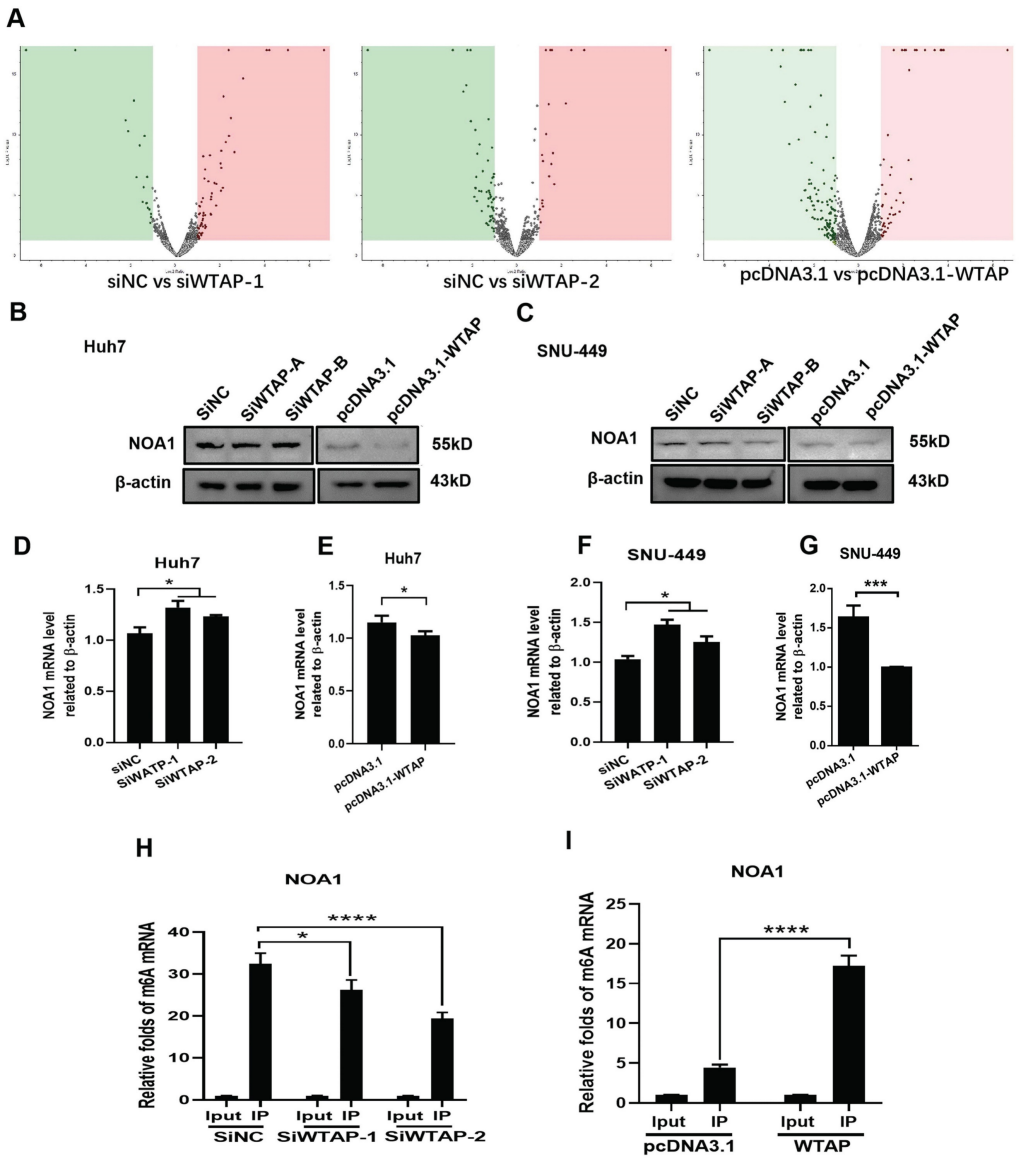
** WTAP affects the m6A methylation of NOA1.** (A) SNU-449 cells were transfected with siRNAs or plasmid using Lipofectamine™ 3000. 48h later, LC/MS was used to analyze the protein expression of NOA1. (B-G) Huh-7 and SNU-449 cells were transfected with the siRNAs or plasmid by Lipofectamine™ 3000. 48h later, total RNA or cell lysates were collected. RT-PCR and Western blot were used to detect the mRNA and protein levels of NOA1. (H-I) SNU-449 cells were transfected with siRNAs or plasmid using Lipofectamine™ 3000. 48h later, MeRIP-qPCR was used to detect the m6A mRNA methylation of NOA1. *p < 0.05, **p < 0.01, *** *p* < 0.001, ****p < 0.001 compared with control groups.

The above data showed that WTAP affected m6A methylation to modulate ferroptosis, resulting in lipid oxidation during the development of HCC. However, the related mechanism remains uncertain. To study the possible regulation mechanism, Western blot and RT-PCR were conducted to analyze the expression of NOA1 during WTAP knockdown or overexpression. DEGs analysis of LC-MS/MS showed that there had sixteen-kinds of differential genes between control groups and WTAP knockdown or overexpression groups (Figure 8A, Figure S1, Table S3). NOA1 is an essential GTPase required for mitochondrial function. Figure 8B-G displayed that NOA1 levels were obviously increased after knockdown of WTAP in Huh7 (*p*<0.05) and SNU-449 cells (*p*<0.05). Nevertheless, overexpression of WTAP could apparently reverse these effects in Huh7 (*p*<0.05) and SNU-449 cells (*p*<0.001). Then, MeRIP-qPCR displayed that knocking-down WTAP apparently inhibited the m6A methylation of NOA1, while overexpressing WTAP could promote the m6A methylation of NOA1, which hinted that WTAP could change the m6A methylation of NOA1 (Figure 8H-I).

**Figure 9:**
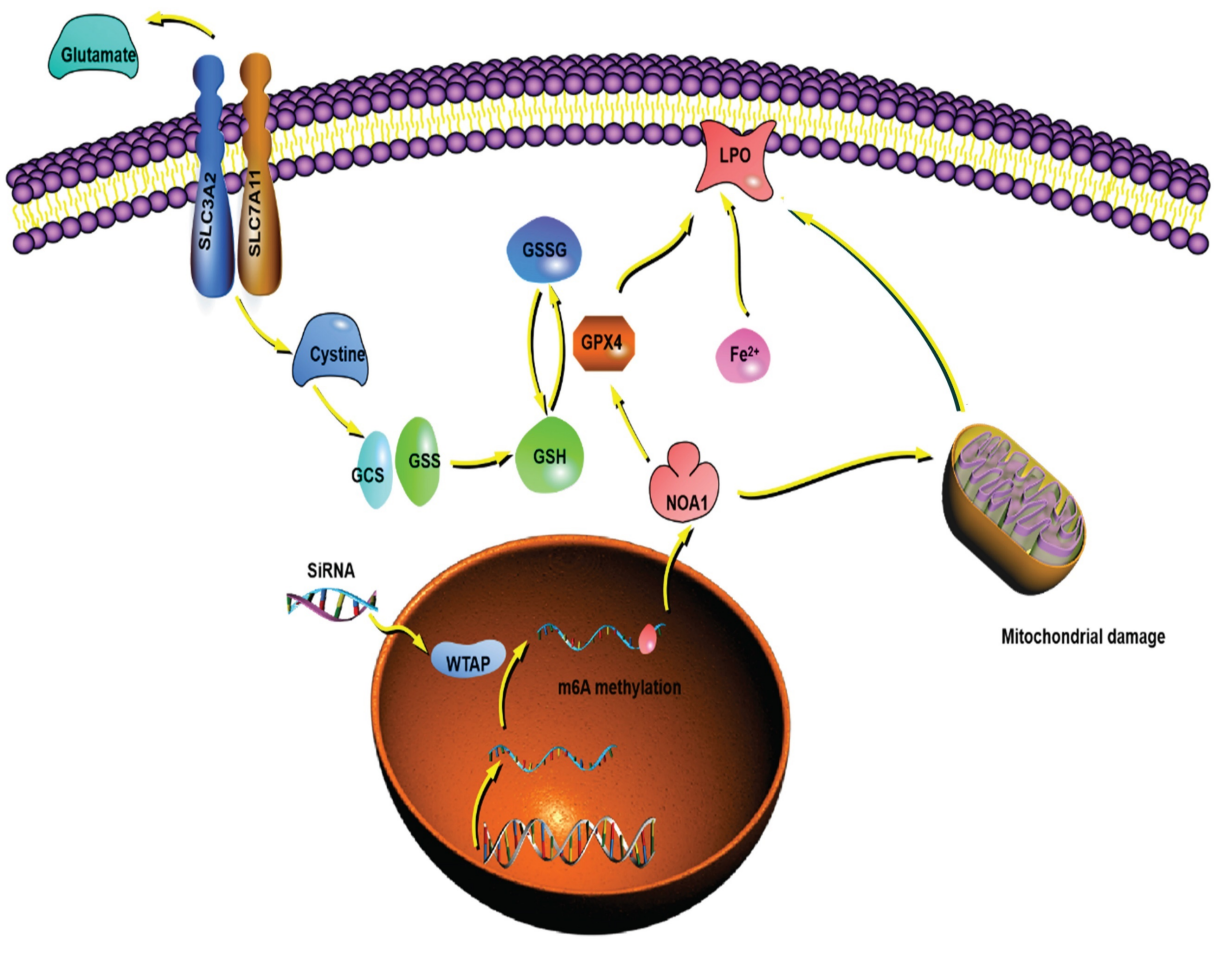
** The putative mechanism of WTAP in this study.** WTAP affects mRNA m6A methylation of NOA1. Lower WTAP finally induces mitochondrial damage, affects GPX4 expression, Fe^2+^ and GSH/GSSH levels to inhibit lipid oxidation, resulting in modulating the pathogenesis and development of HCC.

Overall, we demonstrated that WTAP affected m6A methylation of NOA1, induced mitochondrial damage, activated the GPX4 expression, reduced the Fe^2+^ levels and improved GSH/GSSH levels to inhibit lipid oxidation, which may regulate the occurrence and development of HCC. WTAP can be potentially novel target for the development of an alternative oncotherapy approach (Figure 9).

## Discussion

HCC is one of the most prevalent tumors with poor prognosis and high mortality. Numerous studies expound that N6-methyladenosine (m6A) modification-related genes, including METTL3, METTL14, YTHDC1, YTHDC2, YTHDF1, YTHDF2, WTAP, ALKBH5, FTO, etc., play vital role in the development and radio resistance of many cancers 33, 34. It has been established that m6A RNA methylation is the most prevalent internal modification in mammalian mRNAs and plays critical biological roles by regulating vital cellular processes 35. Dysregulated m6A modification due to aberrant expression of its regulatory proteins is frequently observed in many pathological conditions, particularly in cancer 36. Carcinogenesis is a complex process stemming from genetic and epigenetic change in a specific cell population. Epigenetic change is conventionally known for differential gene expression patterns leading to alternative phenotypic traits, including altered DNA methylation, histone modification, or remodeling of chromatin structure 37. YTHDF1 is confirmed to influence the survival of HCC patients 38.

Current evidence suggests that WTAP regulates ferroptosis in hepatoma cells, which recruits METTL3 and METTL14, promoting the m6A methyltransferase to bind with RNA. Moreover, WTAP regulates gene transcription and RNA processing 39. Another research showed that WTAP is overexpressed in acute myeloid leukemia (AML), inhibiting cell proliferation and colony formation, inducing G1/S-phase arrest. Accordingly, WTAP has huge prospects as a predictor of poor prognosis in AML 40-42. An *in-silico* analysis finds that WTAP significantly affects PI3K/AKT signaling 43. Interestingly, it has been reported that WTAP upregulates the apoptosis ratio of K562 and HL-60 cells treated with etoposide 40. Moreover, WTAP can regulate the dual-specificity phosphatases6 to protect natural killer/T-cell lymphoma (NKTCL) cells from bax-modulated apoptosis induced by cisplatin (DDP) 44. Besides, WTAP promotes renal cell carcinoma proliferation by regulating CDK2 mRNA stability 45. WTAP accelerates the Warburg effect of gastric cancer by regulating HK2 stability 46. WTAP is significantly upregulated in HCC and promotes liver cancer development. WTAP-guided m6A modification contributes to the progression of HCC via the HuR-ETS1-p21/p27 axis 47. Recent studies show that the m6A regulator WTAP can serve as a prognostic biomarker for certain tumors in pan-cancer 48. WTAP deteriorates HCC immune evasion and aerobic glycolysis through decreasing CD8+ T cells' antitumor activity 49. Moreover, it is reported that WTAP deletion in hepatocyte promotes HCC development 50. During the progression and development of HCC, WTAP plays essential role in it 51. In this study, the expressions of WTAP, METTL14, YTHDF3 were inhibited in tumor tissues, the decrease of WTAP levels was most apparent (Figure 1). Consistent with previous studies, WTAP might function as a crucial modulator in the pathogenesis of HCC.

To further confirm this hypothesis, we chose Huh-7 and SNU-449 cells to conduct the experiments (Figure 2). Results suggested that deletion of WTAP in Huh-7 and SNU-449 cells promoted mitochondria dysfunction, which was manifested smaller mitochondria, compressed intermembrane space of mitochondria and lower mitochondrial membrane potential. Overexpression of WTAP could turn on the trend (Figure 3). It hinted that WTAP had a closely connection in mitochondria function of HCC developing. Liu *et al.* demonstrated that WTAP-mediated m6A modification of lncRNASnhg1 regulated myocardial apoptosis, mitochondrial fusion through miR-361-5p/OPA1 axis, resulting in modulating the progression of myocardial-ischemia0reperfusion injury 52. Huang *et al.* also confirmed the role of WTAP in the inflammation, mitochondrial damage and ferroptosis of kidney tubular epithelial cells in acute kidney injury 53. Our study firstly verified the viewpoint about WTAP affecting mitochondrial function in HCC.

As we know, the effectiveness of ferroptosis can be as an anti-tumor therapy approach. Unlike apoptosis, pyroptosis, necrosis, and other forms of cell death, ferroptosis is a form characterized by disrupting the balance of intracellular redox system, inducing lipid peroxidation and ultimately leading to programmed cell death 54. GPX4 is a key enzyme in the ferroptosis signal pathway. It has been established that HCC is related to ferroptosis 55. To further clarify the relationship between WTAP and ferroptosis, flow cytometry was conducted to evaluate the apoptosis. Western blot was used to analyze cell pyroptosis. It was found that WTAP did not affect the positive ratio of Annexin V/PI, which meant that apoptosis wasn't induced by WTAP (Figure 4). Moreover, we detected the expression of the pyroptosis downstream protein GSDMD. No evident difference appeared in the control groups and WTAP deletion or overexpression groups (Figure 5). Interestingly, knockdown of WTAP promoted expressions of GPX4 in hepatoma cells (Figure 6A-F). It was hypothesized that WTAP might affect the rise of ferroptosis in HCC. This conclusion was also verified by Li *et al.* 54. that it is proved that deletion of WTAP or YTHDC2 effectively inhibited ferroptosis and participated in the progress of HCC *in vitro* and *in vivo* models. Other researchers testified the modulation of WTAP on ferroptosis 56-58

Ferroptosis is a Fe^2+^-dependent programmed cell death form characterized by LPO accumulation induced by mitochondrial damage 59. Electron microscopy and the analysis of LPO or Fe^2+^ concentration showed that WTAP deletion caused a significantly increase of GSH/GSSG levels and clearly decrease of Fe^2+^ concentrations appeared in Huh-7 and SNU-449 cells. Accordingly, WTAP overexpression turned on the effect (Figure 6G-R). The effect of WTAP on modulating GSH synthesis might be independent of SLC7A11, not SLC3A2 or the Xc-system (Figure 7). Studies revealed that enhanced lipid catabolism and anabolism for energy and harness lipid metabolism appeared in HCC 60. WTAP had been proved as the regulator of mitochondrial lipid oxidation 61. Based on our data, WTAP deletion could mediate ferroptosis by inducing LPO. This result is similar to previous standpoint.

NOA1 is a vital protein for oxygen-dependent regulation of mitochondrial respiratory complexes and ATP production. It regulated mitochondrial respiration and apoptosis through interacting with DAP3 and complex I. NOA1 deletion trigged oxidative stress and leaded to cell death and showed deficient mitochondrial function and spreading of oxidative phosphorylation 62-65. In this study, the relationship of WTAP and NOA1 had been confirmed by mass spectrometry. Also, qPCR, WB and MeRIP-qPCR were conducted to verify the function of WTAP regulating the m6A methylation of NOA1(Figure 8). It is speculated that during the impact of WTAP on the occurrence and progress of HCC, NOA1 might be a central player in the potential mechanism.

In brief, this work revealed that WTAP may affect the m6A methylation of NOA1 to induce mitochondrial damage, meanwhile activate the GPX4 expression and reduce the Fe^2+^ level, improve GSH/GSSH level to inhibit the lipid oxidation, which may inhibit the occurrence and development of HCC. Regulating the WTAP function will be an alternative oncotherapy method.

## Supplementary Material

Supplementary figure and table.

## Figures and Tables

**Table 1:** Demographics and clinical characteristics of HCC specimens

Characteristics	Value
Age, years	57(40, 75)
Sex, n (%)	
male	16(94.1)
female	1(5.9)
Etiology, n (%)	
HBV	14(82.4)
HCV	0(0)
alcohol	0(0)
others	3(17.6)
Previous HCC treatment history, n (%)	2(11.8)
Cirrhosis, n (%)	9(52.9)
Size of tumor, n (%)	
<5cm	6(35.3)
≥5cm	11(64.7)
Tumor numbers, n (%)	
single	16(94.1)
multiple	1(5.9)
T stage, n (%)	
1-2/3-4	17/0(100/0)
N stage, n (%)	
0/1	17/0(100/0)
M stage, n (%)	
0/1	17/0(100/0)

HBV, Hepatitis B Virus; HCV, Hepatitis C Virus.

## Abbreviations

HCC: hepatocellular carcinoma
WTAP: wilms tumor 1 associated protein
METTL3: methyltransferase 3
METTL14: methyltransferase 14
FTO: fat mass and obesity-associated protein
ALKBH: AlkB homolog
YTHDF1: YTH N6-Methyladenosine RNA binding protein F1
YTHDF2: YTH N6-Methyladenosine RNA binding protein F2
YTHDF3: YTH N6-Methyladenosine RNA binding protein F3
RT-PCR: reverse transcription PCR
MeRIP: methylated RNA immunoprecipitation
ROS: reactive oxygen species
GPX4: glutathione peroxidase 4
NOA1: Nitric oxide-associated protein 1
AMPK: Adenosine 5'-monophosphate (AMP)-activated protein kinase
ACC: acetyl-CoA carboxylase
FSP1: ferroptosis suppressor protein-1
GTP: guanosine triphosphate
OXPHOS: oxidative phosphorylation
LPO: lipid hydroperoxide
PBS: phosphate buffer saline
LC/MS: Liquid Chromatography/Mass Spectrometry
GSDMD: Gasdermin D
AML: acute myeloid leukemia
PI3K: phosphoinositide 3-kinase
CDKs: Cyclin-Dependent Kinases
HK2: hexokinase 2
CCCP: Carbonyl cyanide 3-chlorophenylhydrazone
