# Supplementary material for: WTAP regulates Mitochondrial damage and Lipid oxidation in HCC by NOA1 mediated m6A modification
Source: J Cancer. 2025 Jan 1;16(1):315–30. doi: 10.7150/jca.102618 (PMC11660133; doi:10.7150/jca.102618)

Supplementary material

Journal of Cancer

WTAP regulates Mitochondrial damage and Lipid oxidation in HCC by NOA1 mediated m6A modification

Sheng Liu[1,2#]·Mei Shang[1#]· Jiao Gong[1#]· Hengchang Sun[1]· Bo Hu[1*]

1. Department of Laboratory Medicine, Third Affiliated Hospital of Sun Yat-sen University, Guangzhou 510630, People's Republic of China
2. Guangdong Key Laboratory of Liver Disease Research, Third Affiliated Hospital of Sun Yat-sen University, Guangzhou 510630, People's Republic of China

[#]These authors contributed equally to this work.
[*]Correspondence author: Bo Hu, Department of Laboratory Medicine, Third Affiliated Hospital of Sun Yat-sen University, Guangzhou 510630, People's Republic of China; E-mail: hubo@mail.sysu.edu.cn

Table S1

Table S1 The sequences of siRNA used in this study

**Name**	**Sequence**
siRNA control	Forward: 5’ –UUCUCCGAACGUGUCACGUTT –3’<br>Reverse: 5’ –ACGUGACACGUUCGGAGAATT–3’
siRNA for WTAP-1	Forward: 5’-GGUUCGAUUGAGUGAAACAdTdT-3’<br>Reverse: 5’-UGUUUCACUCAAUCGAACCdTdT-3’
siRNA for WTAP-2	Forward: 5’-AAGCUUUGGAGGGCAAGUAdTdT-3’<br>Reverse: 5’-UACUUGCCCUCCAAAGCUUdGdT-3’

Table S2

Table S2 The sequences of Primers used in this study

Name	Sequence
WTAP	Forward: 5'-ACTGGCCTAAGAGAGTCTGAAG-3'<br>Reverse: 5'-GTTGCTAGTCGCATTACAAGGA-3'
METTL3	Forward: 5'- TTGTCTCCAACCTTCCGTAGT-3'<br>Reverse: 5'- CCAGATCAGAGAGGTGGTGTAG-3'
METTL14	Forward: 5'- AGTGCCGACAGCATTGGTG-3'<br>Reverse: 5'- GGAGCAGAGGTATCATAGGAAGC-3'
FTO	Forward: 5'- GCTGCTTATTTCGGGACCTG -3'<br>Reverse: 5'- AGCCTGGATTACCAATGAGGA -3'
ALKBH	Forward: 5'- GGGAGCACCGAGATGATGAAA-3'<br>Reverse: 5'- CCTTATGCCGGAAGACAAAGT-3'
YTHDF1	Forward: 5'- ACCTGTCCAGCTATTACCCG-3'<br>Reverse: 5'- TGGTGAGGTATGGAATCGGAG-3'
YTHDF2	Forward: 5'- CCTTAGGTGGAGCCATGATTG-3'<br>Reverse: 5'- TCTGTGCTACCCAACTTCAGT-3'
YTHDF3	Forward: 5'- GGTGTATTTAGTCAACCTGGGG-3'<br>Reverse: 5'- AAGAGAACTAGGTGGATAGCCAT-3'

WTAP for pcDNA3.1	Forward: 5'-CCGGAATTCCGATGACCAACGAAGAACCTC-3'<br>Reverse: 5'-CGCGGATCCGCCAAAACTGAACCCTGTA-3'
SiRNA for WTAP-1	Forward: 5’-GGUUCGAUUGAGUGAAACAdTdT-3’<br>Reverse: 5’-UGUUUCACUCAAUCGAACCdTdT-3’
SiRNA for WTAP-2	Forward: 5’-AAGCUUUGGAGGGCAAGUAdTdT-3’<br>Reverse: 5’-UACUUGCCCUCCAAAGCUUdGdT-3’
β-actin	Forward: 5'-CGCGAGAAGATGACCCAGAT-3'<br>Reverse: 5'-CAGAGGCGTACAGGGATAGCA-3'
GPX4	Forward: 5'-GAGGCAAGACCGAAGTAAACTAC-3'<br>Reverse: 5'-CCGAACTGGTTACACGGGAA-3'
SP1	Forward: 5'- GTGGAGGCAACATCATTGCTG -3'<br>Reverse: 5'- GCCACTGGTACATTGGTCACAT -3'
NOA1	Forward: 5'- CCTTCCAGCACTCATCGAGTC -3'<br>Reverse: 5'- TCCAGGATGTACTCCGGGAAC -3'
SLC7A11	Forward: 5'-GGTCCATTACCAGCTTTTGTACG-3'<br>Reverse: 5'-AATGTAGCGTCCAAATGCCAG-3'
SLC3A2	Forward: 5'- TGAATGAGTTAGAGCCCGAGA -3'<br>Reverse: 5'- GTCTTCCGCCACCTTGATCTT -3'

Table S3

Table S3 Mass spectrometry screening results of potential targets of WTAP

Gene	SiWATP-1 vs SiNC (Abundance Ratio)	SiWATP-2 vs SiNC (Abundance Ratio)	pcDNA3.1-WATP vs pcDNA3.1 (Abundance Ratio)
WDR3	2.326488706	2.835728953	0.816530427
TPM3	1.341607565	1.204491726	0.754385965
GADD45GIP1	1.747191011	1.467696629	0.816530427
SP-1	1.322263223	1.367773678	0.686340641
PLIN3	1.415647922	1.251833741	0.808318264
BRD3	1.489270386	1.802575107	0.819836215
HLTF	1.352009744	1.302070646	0.454545455
GTF2F1	1.448979592	1.632653061	0.824817518
TRIP13	1.268907563	1.332533013	0.829826167
MAT2B	1.346200241	1.272617612	0.514004542
UBXN7	1.878333333	2.121666667	0.833180568
SEPHS1	2.579365079	2.371031746	0.790510295
**NOA1**	**1.960948396**	**1.224546722**	**0.501501502**
PPM1G	1.569908815	1.989361702	0.831501832
TLE3	5.327272727	1.236363636	0.418439716
ATPIF1	5.448717949	12.77564103	0.534919417

Figure S1. Venn mapping of different genes between control groups and WTAP knockdown or overexpression groups. LC-MS/MS showed that there had sixteen-kinds of differential genes between control groups and WTAP knockdown or overexpression groups.

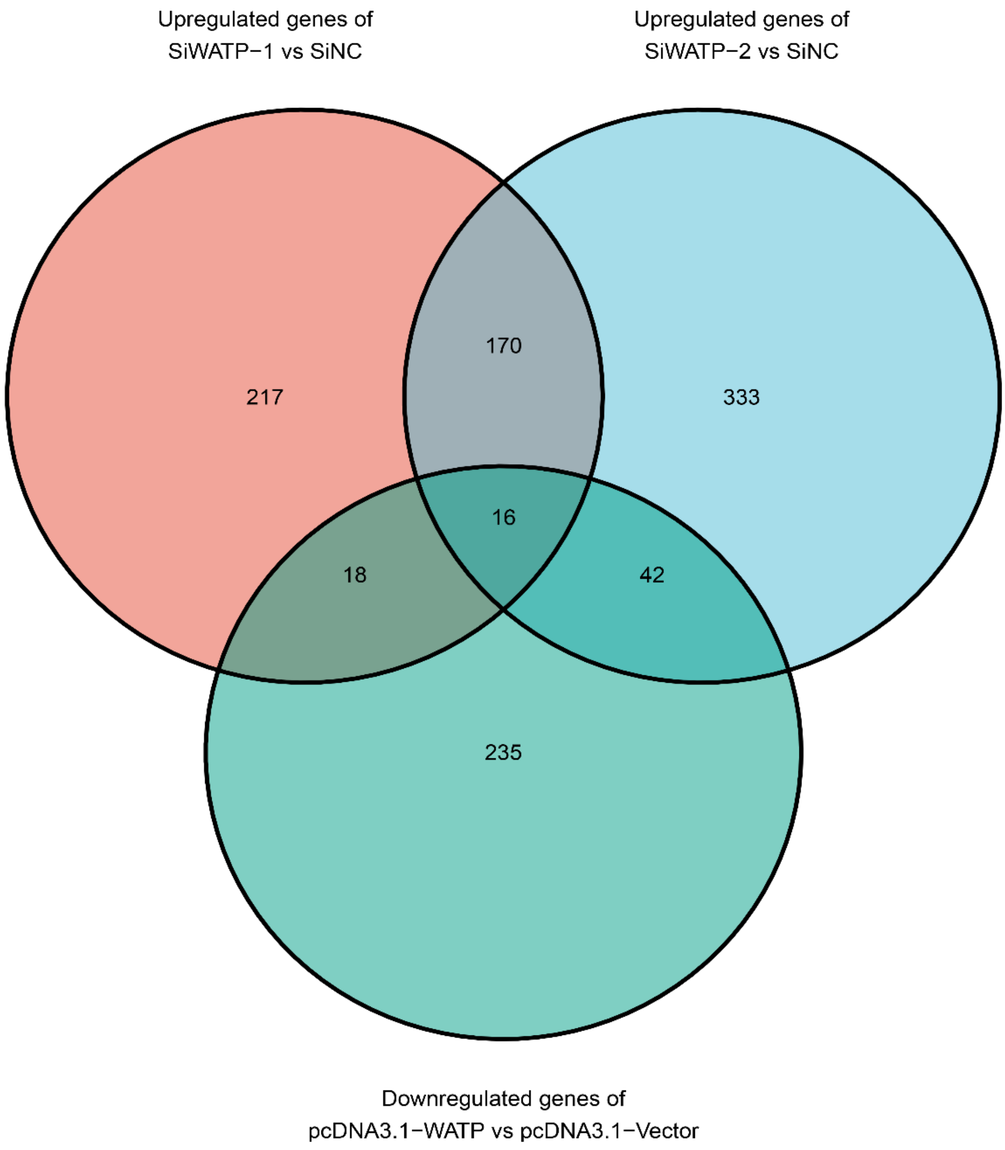

Supplement: Supplementary file 1 — Supplementary figure and table. [file jcav16p0315s1.pdf]
